# Evaluation of P53 and CK20 Immunohistochemical Markers in Comparison with Morphologic Findings in Low- and High-grade Urothelial Carcinomas

**DOI:** 10.30699/IJP.2021.136330.2493

**Published:** 2021-05-09

**Authors:** Mahsa Ahadi, Afshin Moradi, Banafshe Bayat, Hanieh Zham, Seyed Jalil Hosseini, Sara Zahedifar, Afsoon Taghavi

**Affiliations:** Men’s Health and Reproductive Health Research Centre, Shahid Beheshti University of Medical Sciences, Tehran, Iran

**Keywords:** CK20, Grade, P53, Urothelial carcinoma

## Abstract

**Background & Objective:**

Urothelial carcinoma is the seventh most common cancer in the world. The histological classification of papillary carcinoma is one of the most important determinants for its prognosis. Sometimes there is an overlap in the extent of the tumor, and the accurate microscopic diagnosis of the tumor is not always easy. The aim of this study was to evaluate P53 and CK20 immunohistochemical markers in comparison with morphologic findings in low- and high-grade urothelial carcinomas.

**Methods:**

For this descriptive study, urinary bladder samples were collected from 50 cancer patients who had undergone biopsy and surgery in Shohaday-e Tajrish Hospital of Tehran, Iran, during the years 2015-2016. P53 and CK20 were studied, and the demographic and histopathological characteristics of the tumor were also analysed.

**Results:**

The mean age of patients enrolled in this study (48 males and 2 females) was 65.8±11.9. Twenty-five cases presented with low-grade and 25 cases presented with high-grade papillary urothelial carcinomas. Sensitivity, specificity, and positive and negative predictive values for P53 were 48%, 80%, 70.5%, and 60.6%, respectively, while the same values for CK20 were 44%, 92%, 84.6%, and 62.2%, respectively. Immunohistochemical results were also positively correlated with the extent of the tumor.

**Conclusion:**

Based on the results, P53 and CK20 may serve as specific markers for diagnosis of low- and high-grade papillary urothelial carcinoma but not sensitive. P53 and ck20 staining have also a high specificity as 80% and 92% and low sensitivity compared to the low and high morphology of papillary carcinoma, thus their positive and their staining intensity are valuable for diagnosis, but their negative results are not determinant.

## Introduction

Urothelial carcinoma is the seventh most common cancer in the world, with approximately 336,000 new cases being diagnosed each year. Many factors have been known as risk factors of this condition. The most common risk factor is smoking, with the incidence in smokers being 2 to 4-fold higher than that in the general population and a decline of incidence up to 1.9 fold after smoking cessation (1-7). The mechanism of tobacco in the pathogenesis of bladder carcinoma is not known, however various carcinogenic factors have been identified in cigarettes, including acrolein, 4-aminobenzyl, arylamine, and oxygen free radicals (8-12). 

Other risk factors include fried and fatty foods and arsenic-containing water, along with analgesic and urinary tract infection, parasites (schistosomiasis), fungi, bladder stones, pelvic radiation, and chemotherapy drugs such as cyclophosphamide (13). The most common symptom is hematuria. It can be asserted that the incidence of urothelial carcinoma in a patient with gross hematuria and in a patient with microscopic hematuria are 20% and 10%, respectively (14-17). The histological classification of papillary carcinoma is one of the most important determinants for its prognosis. This classification was first introduced by the World Health Organization (WHO) (18-20).

The mentioned classification is based on the grade of anaplasia. Anaplasia itself was defined by another classification proposed by the WHO in the same year as increased cellularity, nucleus accretion, loss of cell polarity and cell differentiation from the basement membrane to the cell surface, nuclei pleomorphism, cell size differences, chromatin pattern, abnormal mitosis, and giant cell (21-24). This classification is one of the grading systems of papillary tumors which is accepted by pathologists, urologists, and oncologists in the United States and elsewhere (25-30). Clinically, the extent of the tumor importantly affects the type of treatment recommended and patient prognosis (1). Sometimes there is an overlap in the extent of the tumor, and the accurate microscopic diagnosis of the tumor is not always easy; hence we tried to find an auxiliary method to confirm what the pathologist observes. The purpose of this study was to accurately determine the tumor grade using the relationship between the immunohistochemical (IHC) expression of P53 and CK20 and the pathological findings including grading and staging of diseases to confirm the pathologist's diagnosis by another method and select a better treatment and do a more successful follow-up.

## Material and Methods

**Study Population**

Patients with bladder cancers who underwent surgical sampling at Shohadaye-Tajrish Hospital of Tehran, Iran, between 2015 and 2016 were the subjects of the study. The sample size in this study was 50 individuals, the same as that in other similar studies.

**Measurements**

**Sample Selection**

We selected 50 patients with papillary urothelial carcinoma, who underwent biopsy in Shohaday-e Tajrish Hospital and referred to Pathology ward. After recording the demographic information, the best samples (in tumor volume) were selected equally (25 low-grade and 25 high-grade cases). IHC staining was done for P53 and CK20 markers and the histopathologic findings were evaluated (obtained from patients’ previous medical records including sex, age, and T-stage).

**Immunohistochemical Staining**

IHC expression of P53 and CK20 was performed. Streptavidin, biotin, and peroxidase (Labeled Streptavidin Biotin / HRP) complex assay was performed on 4-μm cut sections of tissue. After excision and deparaffinization and dehydration in alcohol, to inhibit internal peroxidase activity, the slices were incubated in 3% hydrogen peroxide for 20 minutes followed by antigen retrieval. The cells were incubated at 121°C for 20 minutes. Specific markers were utilized for the staining process. 

Evaluation of immunohistochemical staining

Given the staining intensity and proportion of positive cells (proportion of nuclear and cytoplasmic staining), scoring criteria for P53 and CK20 were considered based on the review of several articles and books on IHC staining. The cut-off points for P53- and CK20-positive or negative were determined as follows:

P53 and CK20 staining was considered positive if more than 5% of tumoral cells were stained. Staining was considered mild if 5-10% of cells were stained, while staining over 50% was indicative of strong staining. All in between were considered as moderate (5).

**Ethical Considerations**

The principles (Ethics fundamental) were considered throughout the study. Researchers also complied with the provisions of the Helsinki during the study, and no additional costs were imposed on patients.

**Statistical Analysis**

Data were analysed by SPSS 23 (SPSS Inc., Chicago, Ill., USA). Categorical data were described as frequency (%) with a 95% confidence interval in 5 normal groups. Chi-square test was also used to analyse the difference between the indices.

## Results

**Fig. 1:**
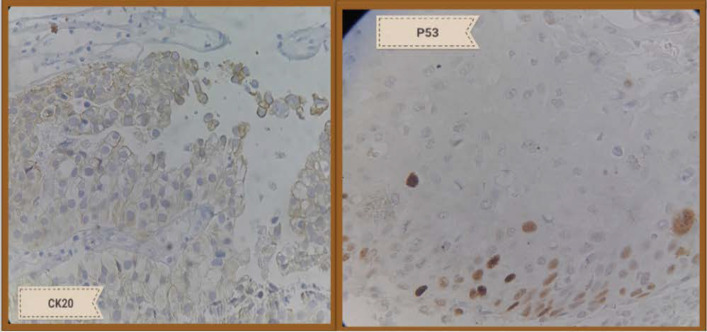
Mild positive staining for CK20 (left side) and P53 (right side)

**Fig. 2:**
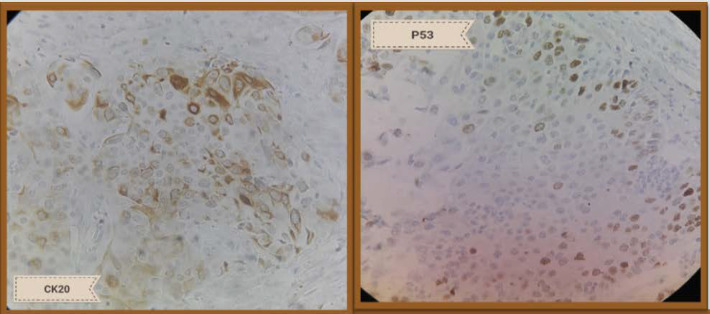
Moderate positive staining for CK20 (left side) and P53 (right side)

**Fig. 3:**
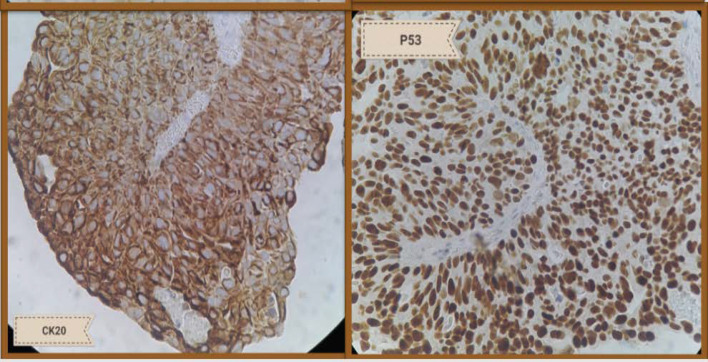
Severe positive staining for CK20 (left side) and P53 (right side)

This descriptive study was conducted on 50 patients with papillary urothelial carcinoma, and tumor specimens of these patients were evaluated for the expression of P53 and CK20 markers. Gender, age, tumor infiltration depth, and histologic grade, as well as the association between each of these markers and the mentioned variables were also investigated. According to the demographic data, 48 out of 50 patients were male while only 2 were female, with a mean age of 65.8±11.9 years. The minimum age was 17 and the maximum age was 82 years (Table 1). In our study, IHC staining for P53 was positive in 52% of low-grade, 80% of high-grade carcinomas and CK20 was immunoreactive in 56% of low-grade and 92% of high-grade cases (Figures 1, 2 and 3).

Based on the morphologic findings for P53 and CK20 staining, the sensitivity, specificity, and positive and negative predictive values of CK20 staining compared to the morphological criteria for the diagnosis of high- and low-grade carcinomas were 44%, 92%, 84.6%, and 62.2%,, respectively, while these findings for P53 were 48%, 80%, 70.5%, and 60.6%, respectively (Table 2). Moreover, the distribution of samples based on the depth of tumor infiltration and IHC expression of P53 and CK20 indicated no significant correlation between the depth of tumor infiltration and staining for CK20 (*P*=0.18) and P53 (*P*=0.38) (Table 3). P53 and CK20 staining showed a high specificity (80% and 92%) and low sensitivity compared to the low and high morphological indices of papillary carcinoma; thus their positive and their staining intensity were valuable for the diagnosis, but their negative results were not determinant and we achieved the main goal to a larger extent.

**Table 1:** Age and gender of the evaluated patients

Variable	Values
Age (Years)	
Mean	65.8
SD	11.95
Gender	
Male	48 (96)
Female	2 (4)

**Table 2:** Comparison of P53 and CK20 immunohistochemical stains and tumour grade

Statistical Values	Grade		IHC
	High	Low	
**Sensitivity: 48** **Specificity: 80** **PPV: 70.5** **NPV: 60.6**			**P 53**
	20	13	**Positive**
	5	12	**Negative**
**Sensitivity: 44** **Specificity: 92** **PPV: 84.6** **NPV: 62.2**			**CK 20**
	23	14	**Positive**
	2	11	**Negative**

**Table 3:** Comparison of P53 and CK20 immunohistochemical stains based on tumor depth

P-value	Tumor Depth					IHC
	T4	T3	T2	T1	Ta	
0.38						**P 53**
	3	1	7	9	13	Positive
	0	0	2	4	11	Negative
0.18						**CK 20**
	3	1	8	11	14	Positive
	0	0	1	2	10	Negative

## Discussion

Bladder cancer is the seventh most common cancer worldwide, and approximately 336,000 new cases are diagnosed each year. Many factors have been known as risk factors of this condition. The most common risk factor is smoking, with the incidence in smokers being 2 to 4-fold higher than that in the general population and a decline of incidence up to 1.9- fold after smoking cessation (1). The mechanism of tobacco in bladder carcinoma is not known, however various carcinogenic factors have been identified in cigarettes, including acrolein, 4-aminobenzyl, arylamine, and oxygen free radicals (3, 4). The aim of this study was to evaluate the IHC expression of P53 and CK20 in comparison with morphologic findings in low- and high-grade urothelial carcinomas.

The evaluation was done on 50 patients with papillary cancer who underwent surgical procedure and/or biopsy at Shohaday-e Tajrish Hospital during 2015-2016. P53 and CK20 immunohistochemical staining were performed and the histopathologic features were evaluated. According to the results, P53 and CK20 stains demonstrated specificity of 80% and 92% for the low- and high-grade papillary carcinomas, respectively, while their sensitivity was 48% and 44%, respectively. In addition, there were significant correlations between positive and negative markers (p53 & CK20) in high- and low-grade tumors, but not between staining and tumor infiltration depth. Other studies of the same field have shown different results that have been discussed in the following. 

In a study by Toll *et al.,* invasive and non-invasive cases of papillary carcinoma were immunohis-tochemically stained for Ki67, P53, E-Cadherin, and CK20. No significant difference was found between the invasive and non-invasive cases regarding IHC study (31). In another study by Roychowdhury *et al*., P53 staining was performed on high- and low-grade papillary carcinoma cases. It was found that the expression of P53 plays an important role (32). Moreover, Anadi *et al.* found that P53 marker was strongly associated with the expression of high- and low-grade tumors (33). Furthermore, Shim *et al.* immunohistochemically analysed the Ki67, P53, and CK20 markers. They observed that tumor grade and IHC results were significantly associated only for Ki67 (34). In addition, Mumtaz *et al.* Performed P53 and CK20 staining in both high- and low-grade papillary carcinomas, where a significant association were reported between tumor grade and IHC results for these two markers (5). In the study of Rajcani *et al., *IHC analyses of Ki67, HMWCK, and P53 in cases of bladder carcinoma and chronic bladder inflammation showed a significant association between tumor grade and Ki67 and HMWCK markers. They also mentioned that P53 was positive in chronic inflammation with pre-malignant changes (3). Finally, we have to say, studies were also performed on other markers and genes, such as Her2 and MDM2 for differentiation between different grades of urothelial carcinoma which, in *Moradi Tabrizi et al*, study no significant association was found between Her2 expression and different degrees of bladder cancer malignancies (35) and study of *Jalali Nadoushan et al.*, overexpression of MDM2 oncoprotein has been shown to be directly related to bladder tumor’s grade (36).

##  Conclusion

Accordingly, most studies in this field have been consistent with the results of our study, indicating that the mentioned factors can be helpful in diagnosing and evaluating each patient prognosis. 

## References

[1] Bertz S, Otto W, Denzinger S, Wieland WF, Burger M, Stöhr R (2014). Combination of CK20 and Ki-67 immunostaining analysis predicts recurrence, progression, and cancer-specific survival in pT1 urothelial bladder cancer. Europ Urol.

[2] Kolahdoozan Sh, Sadjadi A, Radmard AR, Khademi H (2010). Five Common Cancers in Iran. Arch Iran Med.

[3] Hodges KB, Lopez-Beltran A, Davidson DD, Montironi R, Cheng L (2010). Urothelial dysplasia and other flat lesions of the urinary bladder: clinicopathologic and molecular features. Human Pathol..

[4] Ohsawa I, Nishimura T, Kondo Y, Kimura G, Satoh M, Matsuzawa I (2004). Detection of urine survivin in 40 patients with bladder cancer. J Nippon Med Sch.

[5] Bray F, Ferlay J, Soerjomataram I, Siegel RL, Torre LA, Jemal A (2018). Global cancer statistics 2018: GLOBOCAN estimates of incidence and mortality worldwide for 36 cancers in 185 countries. CA Cancer J Clin.

[6] Antoni S, Ferlay J, Soerjomataram I, Znaor A, Jemal A, Bray F (2017). Bladder cancer incidence and mortality: a global overview and recent trends. European urology.

[7] Asgari M, Maybodi MN, Abolhasani M (2016). Differential diagnosis of urothelial carcinoma in situ from non-neoplastic urothelia: Analysis of CK20, CD44, P53 and Ki67. Med J Islam Repub Iran..

[8] Rajcani J, Kajo K, Adamkov M, Moravekova E, Lauko L, Felcanova D (2013). Immunohistochemical characterization of urothelial carcinoma. Bratislavske Lekarske Listy.

[9] Khayamzadeh M, Aliakbari F, Zolghadr Z, Emadeddin M, Ahadi M, Akbari ME, Abedi AR, Nematollahi Sh, Hosseini SJ (2020). Five-year Survival Rate of Bladder Cancer in Iran during 2001-2007. Iran J Pathol,.

[10] Mai KT, Flood TA, Williams P, Kos Z, Belanger EC (2013). Mixed low-and high-grade papillary urothelial carcinoma: histopathogenetic and clinical significance. VirchowsArchiv.

[11] Shariat SF, Ashfaq R, Karakiewicz PI, Saeedi O, Sagalowsky AI, Lotan Y (2007). Survivin expression is associated with bladder cancer presence, stage, progression, and mortality. Cancer: Interdisciplin Int J Am Cancer Soc.

[12] Mumtaz S, Hashmi AA, Hasan SH, Edhi MM, Khan M (2014). Diagnostic utility of p53 and CK20 immunohistochemical expression grading urothelial malignancies. Int Arch Med.

[13] Hasan IA, Gaidan HA, Al-kaabi MM (2018). Diagnostic value of immunohistochemical panel (Cytokeratin CK 7, Cytokeratin CK20, High molecular weight cytokeratin HMWCK (clone CK34βE12) and Prostatic specific antigen (PSA) in differentiation between poorly differentiated prostatic and urothelial carcinoma. Iraq J Cancer Med Gen.

[14] Weyerer V, Schneckenpointner R, Filbeck T, Burger M, Hofstaedter F, Wild PJ (2017). Immunohistochemical and molecular characterizations in urothelial carcinoma of bladder in patients less than 45 years. J Cancer.

[15] Buolamwini JK (2005, Feb). : Small molecule antagonists of the MDM2 oncoprotein as anticancer agents. Curr Cancer Drug Target.

[16] McKenney J, Desai S, Cohen C, Amin MB (2001). Discriminatory Immunohistochemical Staining ofUrothelial Carcinoma in Situ and Non-neoplastic Urothelium. Am J Surg Pathol.

[17] Cumberbatch MGK, Noon AP (2019). Epidemiology, aetiology and screening of bladder cancer. Transl Androl Urol.

[18] Yang Y, Kaimakliotis HZ, Williamson SR, Koch MO, Huang K, Barboza MP (2019). Micropapillaryurothelial carcinoma of urinary bladder displays immunophenotypic features of luminal and p53-like subtypes and is not a variant of adenocarcinoma. Urol Oncol: Seminars and Original Investigations.

[19] Korkolopoulou P The role of p53, mdm2, and b-2 oncoproteins, epidermal growth factor receptor and prolifration markers in the prognosis of urinary bladder cancer. Patol Res Pract.

[20] Das D, Dey RK, Saha S, Das TK (2015). Utility of a dual immunostain like p53 and CK20 to aid in the diagnosis and categorization of neoplastic bladder biopsies. J Evol Med Den Sci-JEMDS.

[21] Barth I, Schneider U, Grimm T, Karl A, Horst D, Gaisa NT (2018). Progression of urothelial carcinoma in situ of the urinary bladder: a switch from luminal to basal phenotype and related therapeutic implications. Virchows Archiv.

[22] Humphrey PA, Moch H, Cubilla AL, Ulbright TM, Reuter VE (2016). The 2016 WHO classification of tumours of the urinary system and male genital organs-part B: prostate and bladder tumours. Eur Urol.

[23] Chou R, Gore JL, Buckley D, Fu R, Gustafson K, Griffin JC (2015). Urinary Biomarkers for Diagnosis of Bladder Cancer: A Systematic Review and Meta-analysis. Ann Intern Med.

[24] Koletsas N, Koletsa T, Choidas S, Anagnostopoulos K, Touloupidis S, Zaramboukas T (2017). Immunohistochemical Investigation of HER/AKT/mTOR Pathway and Cellular Adhesion Molecules in Urothelial Carcinomas. Patholog Res Int.

[25] EL-RASHIDY M, AYA S (2018). Immunohistochemical Study of the Role of CK20, p53 and Ki-67 in Differentiation of Some Urothelial Lesions and Urothelial Carcinoma of the Urinary Bladder. Med J Cairo Uni.

[26] Sanli O, Dobruch J, Knowles MA, Burger M, Alemozaffar M, Nielsen ME (2017). Bladder cancer. Nature reviews Dis primers.

[27] Yikilmaz TN, Dirim A, Ayva ES, Ozdemir H, Ozkardes H (2016). Clinical use of tumor markers for the detection and prognosis of bladder carcinoma: A comparison of CD44, cytokeratin 20 and survivin. Urol J.

[28] Kamat AM, Hahn NM, Efstathiou JA, Lerner SP, Malmström PU, Choi W (2016). Bladder cancer. The Lancet.

[29] (2002,). Uchida t: Clinical significance of p53, mdm2 and bci2 expression in transitional cell carcinoma of bladder. Oncol Rep.

[30] Hashimoto h (2000). Role of p53 and mdm2 in tumor proliferation and determination of the prognosis of TCC of renal pelvises and ureter. Int J Urol.

[31] Toll AD, Epstein JI (2012). Invasive low-grade papillary urothelial carcinoma: a clinicopathologic analysis of 41 cases. The American journal of surgical pathology.

[32] Roychowdhury A, Dey R, Bandyapadhyay A, Bhattacharya P, Mitra R, Dutta R (2012). Study of mutated p53 protein by immunohistochemistry in urothelial neoplasm of urinary bladder. J Indian Med Assoc.

[33] Anadi RC, Dey RK Expression of p53 Protein by Immunohistochemistry in Urothelial Neoplasm. A Hospital-based Study from Eastern India.

[34] Shim J-W, Cho KS, Choi Y-D, Park Y-W, Lee D-W, Han W-S (2008). Diagnostic algorithm for papillary urothelial tumors in the urinary bladder. Virchows Archiv.

[35] Moradi Tabrizi H, Nazar E, Ahmadi SA, Azimi E, Majidi F (2021). Survivin and Her2 Expressions in Different Grades of Urothelial Neoplasms of Urinary Bladder. Iran J Pathol.

[36] Jalali Nadoushan MR, Ghorbanian E, Taheri T (2006). Relationship between grade and MDM2 oncoprotein overexpression in transitional cell carcinoma of the urinary bladder. Iran J Pathol.

